# Properties and Characterization of Lignin Nanoparticles Functionalized in Macroalgae Biopolymer Films

**DOI:** 10.3390/nano11030637

**Published:** 2021-03-04

**Authors:** Samsul Rizal, Tata Alfatah, Abdul Khalil H. P. S., E. M. Mistar, C. K. Abdullah, Funmilayo G. Olaiya, F. A. Sabaruddin, Ikramullah, Umar Muksin

**Affiliations:** 1 Department of Mechanical Engineering, Universitas Syiah Kuala, Banda Aceh 23111, Indonesia; ikramullah@mhs.unsyiah.ac.id; 2 School of Industrial Technology, Universiti Sains Malaysia, Penang 11800, Malaysia; eka.marya.mistar@serambimekkah.ac.id (E.M.M.); ck_abdullah@usm.my (C.K.A.); phunmieoseyemi@gmail.com (F.G.O.); atiyah88@gmail.com (F.A.S.); 3 Department of Physics, Universitas Syiah Kuala, Banda Aceh 23111, Indonesia; muksin.umar@unsyiah.ac.id

**Keywords:** bioplastic, macroalgae, lignin nanoparticles, purification, packaging material

## Abstract

The demand for bioplastic material for industrial applications is increasing. However, moisture absorption and low mechanical strength have limited the use of bioplastic in commercial-scale applications. Macroalgae is no exception to these challenges of bioplastics. In this study, Kappaphycus alvarezii macroalgae were reinforced with lignin nanoparticles. Lignin nanoparticles (LNPs) were used as a filler to reduce the brittleness and hydrophilic nature of macroalgae (matrix). Lignin nanofiller was produced using a green approach from black liquor of soda pulping waste and purified. The physical, mechanical, morphological, structural, thermal, and water barrier properties of LNPs with and without the purification process in macroalgae films were studied. The bioplastic films’ functional properties, such as physical, mechanical, thermal, and water barrier properties, were significantly improved by incorporating purified and unpurified LNPs. However, the purified LNPs have a greater reinforcement effect on the macroalgae than unpurified LNPs. In this study, bioplastic film with 5% purified LNPs presented the optimum enhancement on almost all the functional properties. The enhancement is attributed to high compatibility due to strong interfacial interaction between the nanofiller and matrix. The developed LNPs/macroalgae bioplastic films can provide additional benefits and solutions to various industrial applications, especially packaging material.

## 1. Introduction

The synthetic polymer from fossil fuels has contributed significantly to industrial and technological development. However, the disposal of synthetic polymers has resulted in severe environmental pollution. The rapidly increasing production of permanent waste generated from synthetic polymers and their effect on the environment is now a global challenge. This is mainly due to their non-biodegradability, difficulty in recycling, and contamination. This has adversely impacted humans, wildlife, and the natural environments of wildlife [1]. Therefore, continuous findings have been made to manage synthetic waste by replacing them with eco-friendly alternatives. 

Polymers derived from natural materials are called biopolymers and have been researchers’ focus as an alternative for the non-biodegradable polymer. Biopolymers are derived from natural plants, living organisms, and biological materials [2,3]. Biopolymers are isolated renewable and edible ingredients such as polysaccharides, protein, and lipids. Biopolymers are a suitable alternative source for packaging materials due to their non-toxicity and biodegradability [4,5]. Macroalgae is an abundant source of polysaccharide derivatives such as agar, carrageenan, and alginate [6]. Macroalgae-based biopolymer has been studied by many researchers [4,7]. Macroalgae-based biopolymer films have good oxygen vapor barrier properties, biocompatible, and low deformability. However, macroalgae-based biopolymer material often possesses a relatively poor vapor barrier due to its hydrophilic nature [8]. Macroalgae have been incorporated with hydrophobic nanofiller to overcome this challenge. This combination of the materials has been reported to improve macroalgae film’s mechanical and barrier properties [9].

Lignin has been used as a filler due to its renewable, non-toxic, and biodegradable properties [10]. Lignin has hydroxyl functional groups used in chemical reactions and allows its effective valorization into value-added products [11]. Lignin is a major by-product of bio-refineries and pulping industries. Annually, lignin production is approximately 130 million tons which are mostly converted to energy generation, but less than 2% is used in value-added applications [12]. The physicochemical properties of isolated lignin are dependent on its precursor materials and processing method. This imparts distinct variants in functional groups, elemental composition, and molecular weight. These properties make lignin interact with many polymers and change their wettability, thermal and mechanical properties. Since lignin has advantageous properties, it has been popular as a promising alternative to conventional petroleum-derived materials [13]. Besides, the native function of mechanical support in plants has promoted it to become one of the most popular structural fillers for polymer composites [14].

Recent studies have reported on the use of lignin in micro size as a reinforcement/filler in biopolymer matrix, such as starch [15], protein [16], polylactic acid [17], polyhydroxybutyrate [18], and bio-based polyamide [19]. However, nano-structured lignin as functional nanofiller in Kappaphycus alvarezii macroalgae is not previously reported in the literature. The present study aimed to develop and characterize lignin nanoparticles (LNPs)/macroalgae bioplastic film in this contribution. The macroalgae were incorporated with unpurified and purified LNPs from black liquor (soda pulping waste), and their functional properties were characterized. Several techniques and analysis methods observed the physical, mechanical, morphological, structural, thermal, optical, and wettability properties of prepared bioplastic films. The isolation of lignin from the industrial waste is a major contrition to waste valorization (for example the pulp and paper industry). Since lignin is hydrophobic in nature (thermoplastic behavior), the incorporation of lignin nanoparticle in macroalgae to improve its hydrophobic for industrial applications is a novel challenge.

## 2. Materials and Method

### 2.1. Materials

Green Leaf Synergy Sdn supplied raw Kappaphycus alvarezii macroalgae. Bhd. (Tawau, Sabah, Malaysia). Detailed chemical composition of the macroalgae has been reported in our previous work [20]. Black liquor of empty fruit bunches (EFB) produced from the soda pulping process was collected from the Division of Bioresource, Paper and Coatings Technology, Universiti Sains Malaysia (USM). Other chemicals used in this study are of analytical grade; glycerol (C_3_H_8_O_3_), sulfuric acid (H_2_SO_4_), diethyl ether ((C_2_H_5_)_2_O), cyclohexane (C_6_H_12_), and ethanol (C_2_H_5_OH). The practical grade of these chemicals was obtained from Sigma Aldrich, Selangor, Malaysia. 

### 2.2. Isolation and Purification of Lignin

Lignin was isolated from EFB black liquor with a modified method by Narapakdeesakul et al. [21]. The liquor’s pH was lowered with concentrated H_2_SO_4_ (4.84 M), slowly dripped into the liquor until its pH is 2.0. The precipitate was filtered using Whatman #42 in a Bushnell funnel and washed with hot distilled water until pH ∼7. The slurry of lignin cake (unpurified) in the filter paper was then oven-dried at 60 °C for 24 h. The isolated, dried lignin was purified with chemical reactions process adapted from Abdul Khalil et al. [22]. The sample was stirred in (C_2_H_5_)_2_O to remove fats and fatty acids, followed by C_6_H_12_/C_2_H_5_OH (1:1, *v*/*v*) extraction in the soxhlet apparatus to remove waxes lipids, and tannins. The produced lignin was washed with hot distilled water (70 °C) for 30 min, and vacuum dried at 85 °C for 24 h and labelled as purified lignin.

### 2.3. Preparation of Lignin Nanoparticles

Unpurified and purified lignin nanoparticles were produced using high-energy ball milling (horizontal ball milling) at a rotation speed of 170 rpm for 24 h in an ambient atmosphere. The stainless steel chamber was loaded with a ratio of ball to lignin powder 10:1 (*w*/*w*). The balls were made of stainless steel with a 20 mm × 12 mm × 10 mm diameter. Then, the lignin nanoparticles were oven-dried at 110 °C for 24 h and kept in a zip lock bag to avoid moisture damage.

### 2.4. Characterisation of Lignin Nanoparticles

Transmission electron microscopy (TEM) of the LNPs was conducted with an energy-filtered EFTEM Libra 120-Carl Zeiss instrument, Selangor, Malaysia. Particle size and zeta potential measurements of LNPs were performed using dynamic light scattering (DLS) on a Malvern Zetasizer Nano ZS Ver. 7.11 (Malvern Instruments, Malvern, UK). Samples were irradiated with red light from HeNe laser, fitted with a wavelength λ = 633 nm. Besides, chemical functional groups of LNPs were observed via a spectrum FT-IR Prestige-21 spectrophotometer (Shimadzu, Chiyoda-ku, Tokyo, Japan).

### 2.5. Fabrication of Lignin Nanoparticles/Macroalgae Composite Films

The solution casting method was used to produce the biocomposite films. The clean macroalgae were soaked in distilled water and cut into small pieces. It was then oven-dried at 40 °C for 72 h before storage [23]. The macroalgae were employed as a base matrix in this study. Five grams of dried macroalgae were dissolved in 250 mL distilled water and glycerol (50% *w*/*w* macroalgae) as a plasticizer in the beaker. Unpurified and purified LNPs were added at a different percentage of loadings (0%, 1%, 3%, 5%, 7%) with reference to the dry weight of macroalgae. The solution was heated to 90 °C for 60 min with continuous stirring and left to settle down till room temperature. The solution was then cast in a 20 cm × 20 cm square area tray and dried in a ventilated oven at 40 °C, 50% relative humidity for 24 h. Five replicates of each sample were cut out for characterization. 

### 2.6. Characterization of Lignin Nanoparticles/Macroalgae Composite Films

#### 2.6.1. Physical Properties

The biocomposite’s physical properties were evaluated by measuring its thickness, moisture content, and moisture absorption capacity. A micrometer screw gauge (Mitutoyo, Kanagawa, Japan) was used to measure the films’ thickness to 0.001 mm or 1 µm precision at 10 random spots on every single film. The corresponding values were taken in the mechanical tests. The average thickness of the films of this study ranged from 101.87 ± 1.77 µm to 119.33 ± 2.75 µm. 

Moisture content (MC) of the composite film was determined by calculating the weight difference before and after drying. The samples of 1 × 1 cm in size were conditioned at 105 °C in the oven for 24 h. Initial (Mi) and final weight (Mf) were measured in four decimal places. Five replicates of each sample were weighed, and the mean values were calculated. The percentage of moisture content was determined with Equation (1).

(1) $$\mathrm{Moisture}\;\mathrm{content}\;(\%)\;=\;\frac{\mathrm{Mi}-\mathrm{Mf}}{\mathrm{Mi}}\;\times \;100$$

The moisture absorption capacity (MAC) of films was measured using Ghanbarzadeh and Almasi [24] method using Equation (2). The dried film of 3 cm × 3 cm was oven-dried at 105 °C until constant weight (W1). The film’s constant weight was measured, and the film was put in a desiccator with distilled water for 48 h at 100% RH and room temperature. The sample was taken out after 48 h and weighed (W_2_). The average of five samples was calculated and used in Equation (2).

(2) $$\mathrm{Moisture}\;\mathrm{absorption}\;\mathrm{capacity}\;(\%)\;=\;\frac{W2-W1}{W1}\;\times \;100$$

#### 2.6.2. Mechanical Properties

Tensile strength (TS), Young’s modulus (YM), and elongation at break (EAB) were determined at room temperature based on the American Society for Testing and Materials (ASTM) D-882-02, 2002 [25]. Five replicates of rectangular 10 mm × 150 mm dimensions were cut for each film. The samples were conditioned in a desiccator at 23 °C and 50% RH for at least 48 h before testing. The analysis was conducted using a tensile strength tester (LLOYD Instruments, Segensworth Fareham, UK) with a capacity of 500 N, and an Initial gauge length of 100 mm was used. The applied rate of grip separation was set at 50 mm/min. 

#### 2.6.3. Surface and Fractured Surface Characterizations by Field Emission Scanning Electron Microscope (FESEM)

Morphological analysis of the LNPs filler loadings compatibility with macroalgae matrix was observed under a scanning electron microscope. Surface and fractured surface morphologies of films were observed with FESEM (FEI Quanta FEG 650, Thermo Fisher Scientific, Eindhoven, The Netherlands). A 1 cm × 1 cm size sample was mounted on an aluminum (Al) stub holder with double-sided copper (Cu) tape holder. The films were coated with a platinum (Pt) layer using sputter and coater Quorum Technologies Q150T to enhance their electrical conductivity. The FESEM micrographs were examined at an accelerating voltage of 5 kV under conventional secondary electron imaging stipulations.

#### 2.6.4. Structural Analysis (FT-IR)

Chemical functional groups were studied using a spectrum FT-IR Prestige-21 spectrophotometer (Shimadzu, Chiyoda-ku, Tokyo, Japan) in the zinc selenide attenuated total refraction procedure (ATR) cell in an infrared spectrometer. Films were prepared and cut with a dimension of 10 mm × 10 mm and oven-dried at 60 °C for 24 h before FT-IR testing. The spectra’ measurement was conducted in transmittance mode, and a wavenumber between 400 to 4000 cm^−1^ for each film was recorded.

#### 2.6.5. Thermal Properties

The thermogravimetric analysis (TGA) of the bioplastic film samples was performed using Mettler-Toledo thermogravimetric analyzer model TGA/DSC 1 (Mettler Toledo, Schwarzenbach, Switzerland). A mass range of 5 to 10 mg biocomposite film was weighed in an alumina crucible and put in a thermogravimetric analyzer with a pre-weighed empty alumina crucible as a reference. The thermal analysis was done at a temperature range of 30 °C to 800 °C, a heating rate of 10 °C/min under nitrogen (N_2_) with a 50 mL/min flow rate. The TG data were interpreted and derived through STAR^e^ SW 10.00 software program to investigate the onset temperature (T_on_) and maximum temperature (T_max_) of decomposition as well as the mass loss (%).

#### 2.6.6. Color and Opacity Properties

Data Color 400 Bench-Top Spectrophotometer was used to determine the color and opacity of the films. Data Color Match software 4.0 (Data color International, Lawrenceville, GA, USA) was used to interpret the data according to the Commission Internationale de l’Elcairage (CIE) L*, a*, b* color system; where L* is lightness ( black to white), a* and b* are the chromatic coordinate (−*a*: greenness, −*b*: blueness, +*a*: redness, +*b*: yellowness). The equipment was initially calibrated to obtain the background (L* = 93.28, a* = −0.13, *b* = 6.47). The film was subjected to the white surface plate with the software to obtain color coordinates. The average of three (3) per sample was calculated. The total color difference (Δ*E*) was calculated according to Equation (3). The opacity of films was determined as a contrast percentage ratio between each film’s opacity with respect to black standard and white standard. The results were computed in percentage (%).

(3) $$\Delta E\;=\;\sqrt{{\left(L^{*}\;-L\right)}^{2}+\;{\left(a^{*}\;-\;a\right)}^{2}+{\left(b^{*}\;-\;b\right)}^{2}}$$

#### 2.6.7. Wettability Analysis

The contact angle was measured with the sessile drop method on KSC CAM 101 (KSV Instruments Ltd., Espoo, Finland) at room temperature. A syringe with approximately 5 µL of water was released to the film surface. Images were immediately shot and documented after the water was dropped onto the film surface. Five measurements were recorded at different positions on the films to determine the average.

#### 2.6.8. Statistical Analysis

The properties of the biocomposite were evaluated with one-way ANOVA using DSAASTAT ver.1.101 by Andrea Onofri. The comparisons test was done using Tukey’s HSD, and the significant differences (*p* < 0.05) between mechanical and physical properties of biocomposite films were determined.

## 3. Results and Discussion

### 3.1. Characterisation of Unpurified and Purified Lignin Nanoparticles

**Figure 1:**
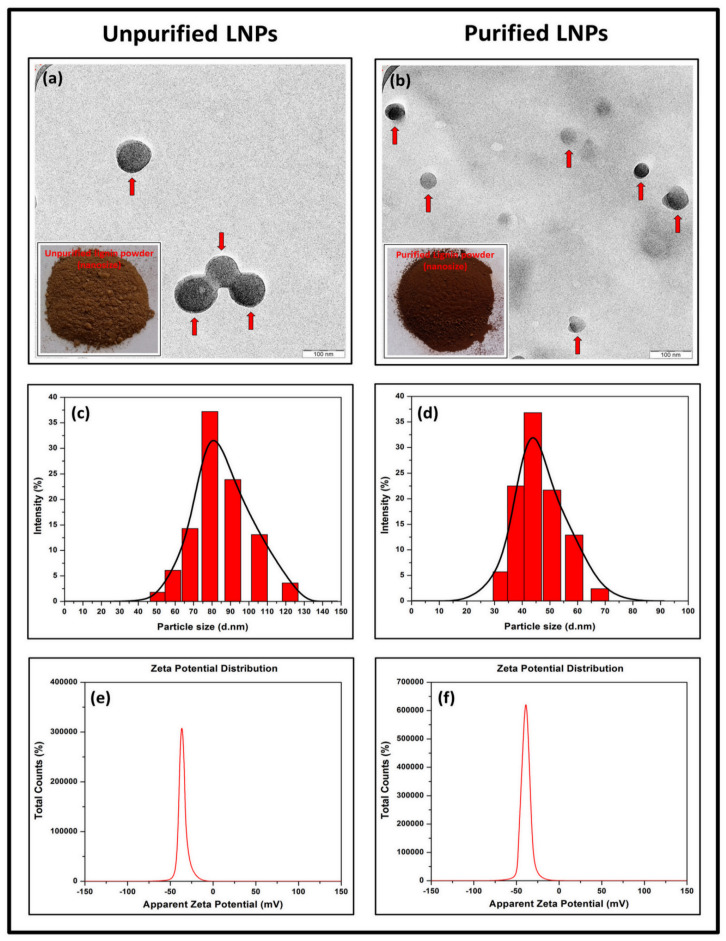
TEM micrograph and nanosized lignin powder (inset figure) of (**a**) unpurified lignin nanoparticles (LNPs) and (**b**) purified LNPs, particle size distribution of (**c**) unpurified LNPs (**d**) purified LNPs, zeta potential distribution of (**e**) unpurified LNPs and (**f**) purified LNPs.

The morphological properties of lignin nanoparticles (LNPs) were analyzed with TEM image J software measurement. Figure 1a,b shows TEM micrographs of LNPs. As seen from the figure, unpurified and purified LNPs possessed irregular size and were uniformly distributed around the range 80 nm to 93 nm and 39 nm to 61 nm, respectively. This similar trend is also reported by Si et al. [26] with lignin. Unpurified LNPs dry powder has bright brown coloration because it contains natural impurities such as fatty acids, waxes, lipids, and tannins. However, purified LNPs in this study have dark brown rich in phenolic compounds [27]. This deeper color was obtained after the purification process [28]. As shown in the inset figure of Figure 1a, a high natural impurities content of unpurified LNPs induces water uptake and self-aggregation of powder, which is not the case with the purified LNPs (inset figure of Figure 1b). Furthermore, the particle size distribution of LNPs in solution was determined by DLS. Figure 1c,d depicts the diameter of unpurified LNPs ranging from 51 nm to 122 nm with an average size of 83.6 nm, meanwhile the diameter of purified LNPs range from 33 nm to 68 nm with the average size of 46.7 nm.

Zeta potential is an assessment of the surface charge of particles to specify the electrostatic interaction between them. It can be measured by evaluating the increasing rate of negatively or positively charged particles through an electric area. The measurement provides pivotal information concerning a colloidal system’s stability with an estimation as a surface charge. It is highly relevant in studying the preparation of nanoparticles [29]. Adequate electrical double layer repulsion between the particles is indicated on high zeta potential (either negative or positive value), which hinders their aggregation [30]. As the effect of the negatively charged phenols and adsorption of hydroxyl ions (to a certain extent), the LNPs commonly have negative zeta potential values [31,32]. Herein, this study evaluated the zeta potential on lignin after the isolation and purification processes. As a result, zeta potential values of unpurified and purified LNPs were negative. As shown in Figure 1e,f, the latter had a higher negative value (−38.2 ± 5.19 mV) compared to the former (−34.4 ± 5.70 mV). It seems that the purification technique leads LNPs to have a more stable surface charge, which was partly owing to the adsorption of hydroxyl ions on the hydrophobic area of the LNPs and partially due to the true negative charges of the phenol groups in the LNPs [33]. Overall, all LNPs showed good stability because the zeta potential values were below −30 mV. This value confirms sufficient mutual repulsion resulting in colloidal or emulsion stability, which can be an essential aspect of enhancing the LNPs-based composites’ properties. 

**Figure 2:**
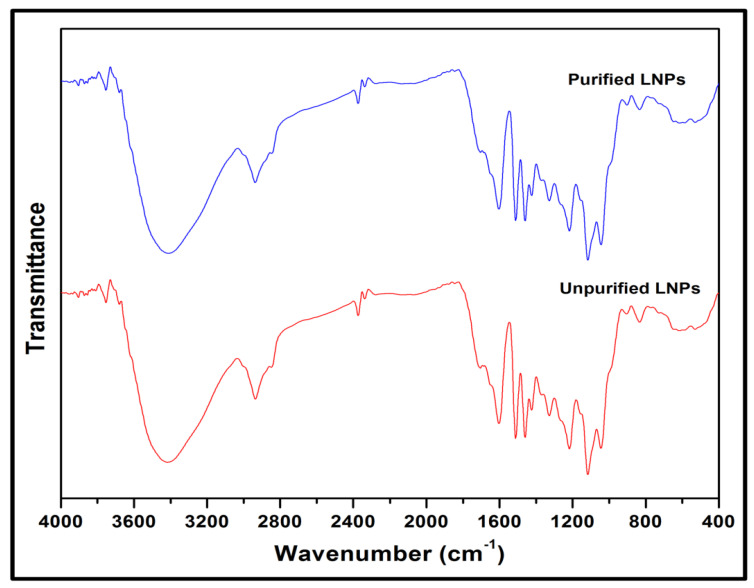
FT-IR spectra of unpurified LNPs and purified LNPs.

The functional groups of unpurified and purified LNPs were investigated using FT-IR with transmittance (%) versus the spectra band (cm^−1^). Figure 2 displays the spectra of absorption bands. Nine absorption major peaks of both LNPs were noticed at 3415, 2938, 2374, 1602, 1512, 1460, 1217, 1116, and 833 cm^−1^. The peaks about 3400–3600 cm^−1^ were associated with O-H stretching vibration of hydroxyl groups. This indicated that the LNPs were bound with some water molecules. Another absorption band around 2938 cm^−1^ was ascribed to the asymmetric C-H representing stretching vibration in methylene and methyl groups. A peak at around 2374 cm^−1^ assigned the presence of C≡C (alkynes) stretching vibration. The band at about 1602 cm^−1^ indicated the presence of C=C stretching vibration in carboxyl, lactone, aldehyde, and ketone. The presence of C=O aromatic structure stretching vibration showed transmittance at approximately 1512 cm^−1^, and the intensity at around 1460 cm^−1^ could be associated with the stretching vibrations of C-H (methyl and methylene). The band’s vibrations attributed to syringyl (1217 cm^−1^) and guaiacyl (1116 cm^−1^) units. Later on, a peak at about 833 cm^−1^ was identified to the structure of aromatic C-H out-of-plane bending vibration.

### 3.2. Characterization of Lignin Nanoparticles/Macroalgae Composite Films

#### 3.2.1. Physical Properties

**Figure 3:**
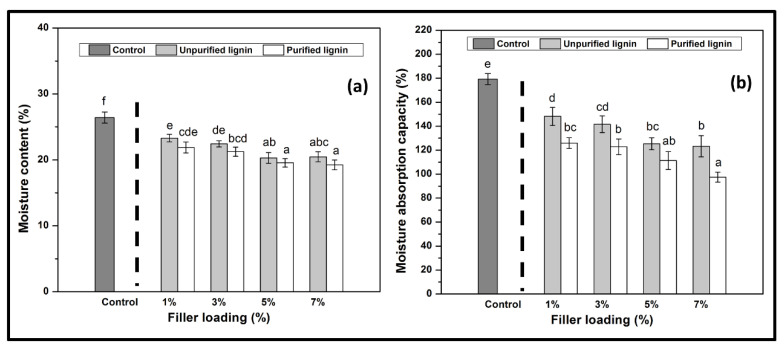
Physical properties of (**a**) moisture content and (**b**) moisture absorption capacity of LNPs/macroalgae biopolymer films. Mean values are plotted with one standard deviation error bar. The letters (a, ab, abc, b, bc, bcd, cd, cde, d, de, e) above data bars indicate no significant difference in the values (*p* < 0.05) analysed by ANOVA test.

**Figure 4:**
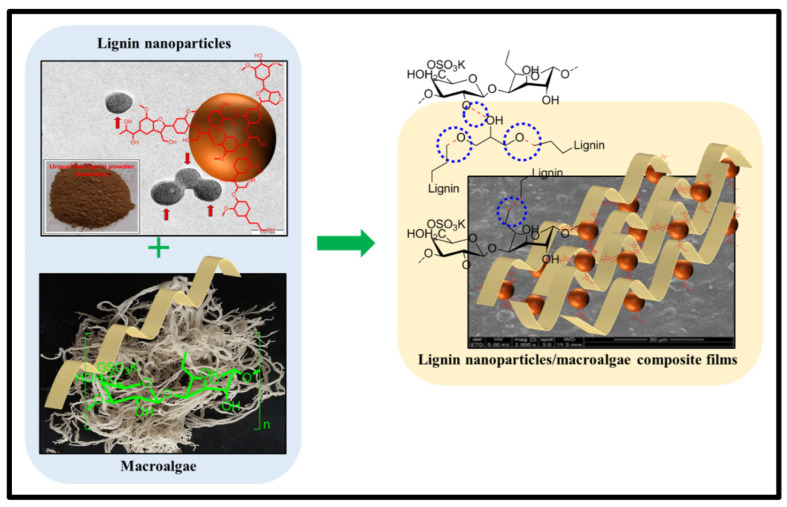
Schematic diagram of LNPs functioned in macroalgae matrix with a blue circle representation of the hydrogen bond.

Figure 3 depicts moisture content (MC) and moisture absorption capacity (MAC) of *Kappaphycus alvarezii* macroalgae reinforced with unpurified and purified LNPs with different loadings. A pure polysaccharide is sensitive to moisture and water owing to its hydrophilic nature. Blending LNPs with macroalgae significantly reduced the resulting film’s MC and MAC percentage. The incorporation higher percentage of LNPs in macroalgae, resulted in a further decrease from 26.42 ± 0.84% to 20.46 ± 0.75% (unpurified LNPs) and to 19.24 ± 0.75% (purified LNPs), and from 179.25 ± 4.67% to 123.24 ± 8.85% (unpurified LNPs) and to 97.49 ± 4.22% (purified LNPs), respectively. This decrease in MC and MAC of the obtained films with filler loading was due to the partial miscibility of hydrophobic phenolic compounds of lignin and the strong formation of hydrogen bonding with the matrix [34]. A schematic illustration of LNPs interactions with macroalgae matrix incorporated after the cast is displayed in Figure 4. 

Although there was a decrease in the MC and MAC properties of films after introducing LNPs as reinforcement filler, the purified LNPs-based films presented a more reduction. The purified LNPs showed lower MC and MAC values than the unpurified LNPs and macroalgae film itself. The reduction of MC and MAC means enhanced hydrophobicity, which is good for film functionality. This indicated that purified LNPs and the macroalgae achieved a high degree of compatibility. The hydrogen bond was probably formed between the higher hydroxyl groups of phenolic compounds in purified LNPs that resulted in the improved compatibility in the matrix molecules [16]. The possible occurrence of interfacial interaction between purified LNPs and matrix had stronger prevention of the water accessibility, which resulted in the ability of the prepared films to absorb moisture. Aqlil et al. [15] reported a similar bioplastic behavior based on lignin when introducing it as a filler in starch-based bionanocomposites.

#### 3.2.2. Mechanical Properties

Potential biopolymers’ mechanical properties are important as their water barrier properties, especially in packaging applications, to their functionality. Mechanical properties present significant information on the brittleness and stiffness of the composite films. The obtained films are expected to resist deformation, depending on their application [35]. Tensile tests were performed to determine the effect of both types of LNPs on the prepared films’ mechanical properties. 

**Figure 5:**
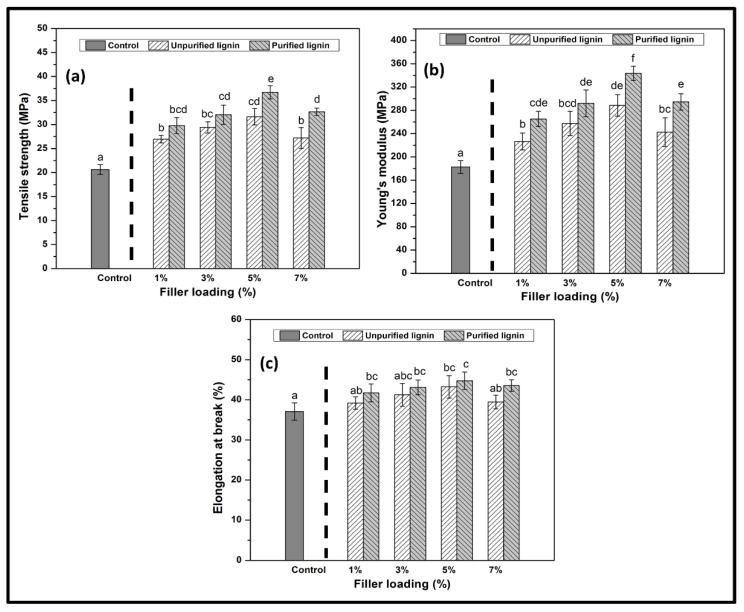
Tensile strength (**a**), Young’s modulus (**b**), and elongation at break (**c**) of LNPs/macroalgae biopolymer films. The letters (a, ab, abc, b, bc, bcd, cd, cde, d, de, e) above data bars indicate no significant difference in the values (*p* < 0.05) analysed by ANOVA test.

Figure 5 exhibits the mechanical properties, namely tensile strength (TS), Young’s modulus (YM), and elongation at break (EAB) of pure macroalgae films, unpurified and purified LNPs-reinforced macroalgae composite films. It was observed that the neat film showed the lowest mechanical properties. The addition of LNPs subsequently improved the mechanical properties of macroalgae-based films. Introduction of 1% unpurified and purified LNPs provided significant enhancement (*p* < 0.05) of TS and YM, for approximately 30.64 and 44.30%, respectively. However, the optimum TS and YM values were observed for 5% purified LNPs of 36.70 ± 1.38 MPa and 343.59 ± 12.32 MPa, respectively. This enhancement in mechanical properties may arise from the greater dispersion in the lignin nanostructures blend, which has a high surface area [36]. Plasticizers enhance the miscibility between hydrophobic lignin nanofillers and hydrophilic macroalgae molecules. The plasticizer enhances the interfacial miscibility of the two polymers [37]. The strong intermolecular interaction of hydroxyl groups between the LNPs and macroalgae enhances its mechanical properties [15].

The decrease of TS and YM after optimum loading was probably due to the increased agglomeration point. The agglomeration point produced stresses which weaken the intermolecular interaction between nanofillers and macroalgae matrix. A similar TS and YM trend was reported in polylactic acid-based composite film reinforced with kraft and acetylated kraft lignins [38]. Overall, purified LNPs fillers probably imparted greater strength compared to those without experiencing the purification process. This result is attributable to the higher content of phenolic compounds contained in purified LNPs than unpurified LNPs.

Values are presented as mean with one standard deviation error bar. The different superscript letters between data bars represent significant differences (*p* < 0.05).

**Figure 6:**
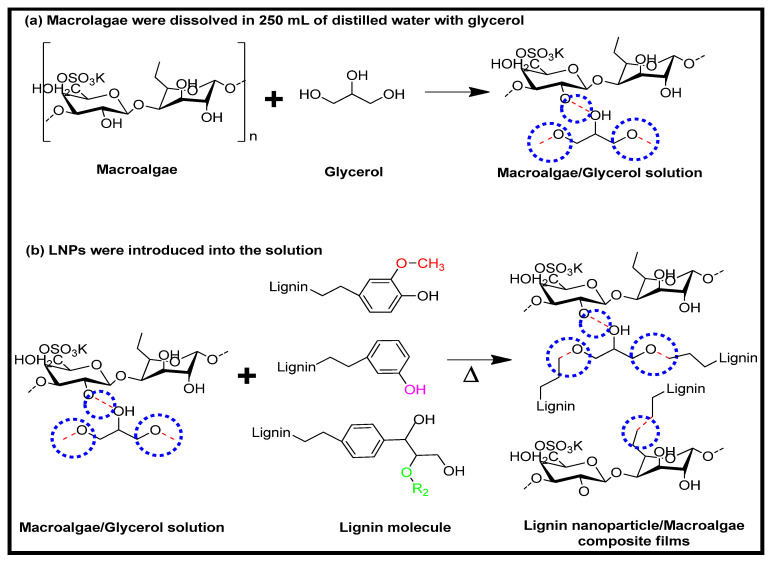
Proposed chemical reaction mechanism of macroalgae biocomposite film functioned with LNPs. The blue circle represents the hydrogen bond. The red circle represents a free available oxygen atom to form a hydrogen bond.

The elongation result describes the flexibility of the composite films. Elastic and flexible plastic product’s ability is significant in applications such as food packaging, agriculture, and cosmetics [39,40,41]. The data showed that the incorporation of 1% of the nanofillers provided significant improvement (*p* < 0.05) of elongation at break (EAB) values. The difference in loading on the macroalgae films increased the EAB values from 37.08 ± 2.15% to 44.76 ± 2.16%. It was observed that the composite films showed greater elongation than the control film. The biocomposite’s elongation gradually increases with the loading of unpurified and purified LNPs from 1% to 5%. However, the elongation of the film decreased by 7% nanofiller. The addition of LNPs at high concentrations (7%) could probably interrupt the macroalgae-glycerol (plasticizer) bonding and eventually decreased the elasticity and flexibility of the composite films. Besides, this observation also agreed with the nanocomposite hydrogel reinforced by biorenewable lignin nanoparticles [40]. The mechanism of possible chemical reaction of macroalgae matrix and glycerol with LNPs is illustrated in Figure 6.

#### 3.2.3. Fracture Morphology Studies

**Figure 7:**
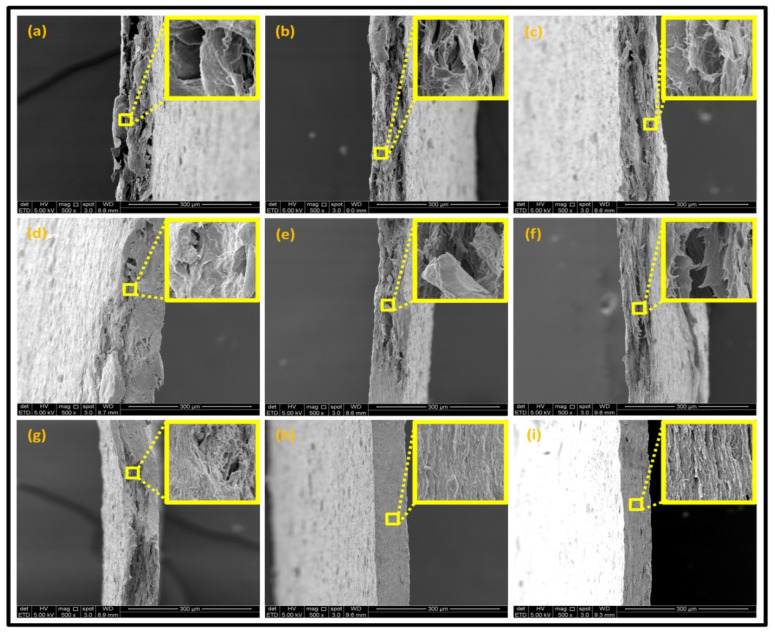
Fracture morphology of (**a**) control film, macroalgae-based biopolymer film with incorporation of unpurified LNPs of (**b**) 1%; (**c**) 3%; (**d**) 5%; and (**e**) 7%, and with addition of purified LNPs of (**f**) 1%; (**g**) 3%; (**h**) 5%; and (**i**) 7%.

Figure 7 displays the fracture surfaces of macroalgae-based films incorporated with various percentages of unpurified and purified LNPs after the mechanical performance. The fracture surface images of films filled with LNPs presented a more organized and layered internal morphology than the film without filler. The fracture surface of films containing unpurified LNPs showed lesser waves with increased nanofiller from 1% to 5% (Figure 7b–d). Interestingly, the waves were also noticeably lesser when the purified LNPs loading increased except for 7% (Figure 7f–h). This was probably due to the uniform shape and smooth surface of purified LNPs; thus, it enhanced the water barrier and hydrophobicity properties. However, the internal morphology was observed of films filled with purified LNPs that provided more structured and lesser waves than the unpurified LNPs and control films. 

Furthermore, the pure macroalgae film exhibited the most waves, which indicated its brittle properties (Figure 7a). The properties aligned with the low mechanical properties of the control film reported in the previous section. The formation of voids and cracks displayed for samples added with a low percentage of LNPs indicates the failure of the LNPs filler to absorb stress during the fracture. As shown in the figure, a lower LNPs filler triggered the crack to propagate and form voids and cavities. However, as the filler percentage increased, particularly as 5%, the biopolymer films showed a smoother surface indicate the optimum filler loading to absorb the stress and prevent crack formation. Better surface properties were observed for film added with purified LNPs, particularly at 5%, attributed to the good compatibility between purified LNPs and macroalgae matrix. It could be concluded that the purified LNPs had better compatibility owing to the formation of high intermolecular interaction between the nanofillers and the macroalgae, which resulted in greater interfacial stress transfer, as also supported by the mechanical analysis. Yang et al. suggested a similar finding of glutaraldehyde crosslinked polyvinyl alcohol films filled with lignin nanoparticles [41]. In this study, the addition of LNPs at 7% led to brittle properties of films and voids’ formation. A similar trend showed for both nanofillers, as shown in Figure 7e,i. This phenomenon may be caused by the saturation of the filler that may impart brittle properties to the film. This finding supported the weak mechanical properties reported where the TS, YM, and EAB of the films decreased with the addition of 7% of the nanofillers. 

#### 3.2.4. Structural Analysis by FT-IR

**Figure 8:**
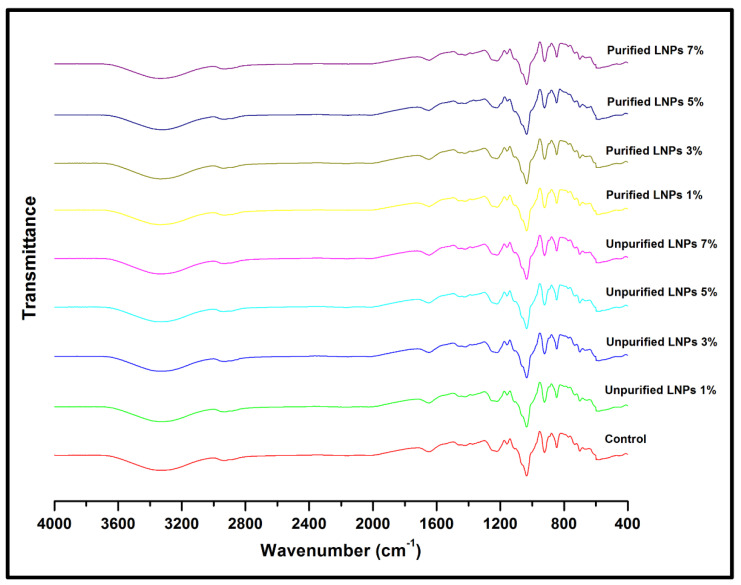
FT-IR spectra of macroalgae, control film and bioplastic films filled with unpurified and purified LNPs.

Figure 8 presents the infrared spectra of the raw macroalgae, macroalgae/glycerol (control), and LNPs/macroalgae composite films. 

According to the FT-IR spectrum for the control film (macroalgae), the broad peak observed at about 3331 cm^−1^ was assigned to the hydroxyl groups’ presence (O-H) of macroalgae [6]. The absorption peak shifted to a lower wave number for the bioplastic film filled with unpurified and purified LNPs. The lower wavenumber of purified LNPs bioplastic films (especially at 5%) compared with pure macroalgae film indicates molecular interaction changes between macroalgae and purified LNPs. The band at 2926 cm^−1^ was ascribed to the C-H stretching. A band at about 2369 assigned the presence of C≡C (alkynes) stretching vibration, which solely appeared from the macroalgae sample. Meanwhile, a peak at approximately 1647 cm^−1^ indicates carbonyl groups (C=O) stretching. This is referred to as the stretching of carboxyl groups in the sulfated polysaccharides of macroalgae [42]. A peak at about 1221 cm^−1^ is attributed to the sulfate ester-stretching of the kappa-carrageenan backbone, which confirmed the presence of carrageenan sulphated polysaccharide. This group represented gelling properties of macroalgae. A band that appeared at about 1036 cm^−1^ for raw macroalgae indicated the glycosidic linkage in all carrageenan types. The peaks at about 924 cm^−1^ and 847 cm^−1^ were assigned to the presence of 3,6-anhydrous-D-galactose (DA) and D-galactose-4-sulfate (G4S), respectively [20]. The FT-IR spectrum of bioplastic films in this study contains the characteristic bands of macroalgae and LNPs as well as the interaction between them. 

#### 3.2.5. Thermogravimetric Analysis

**Figure 9:**
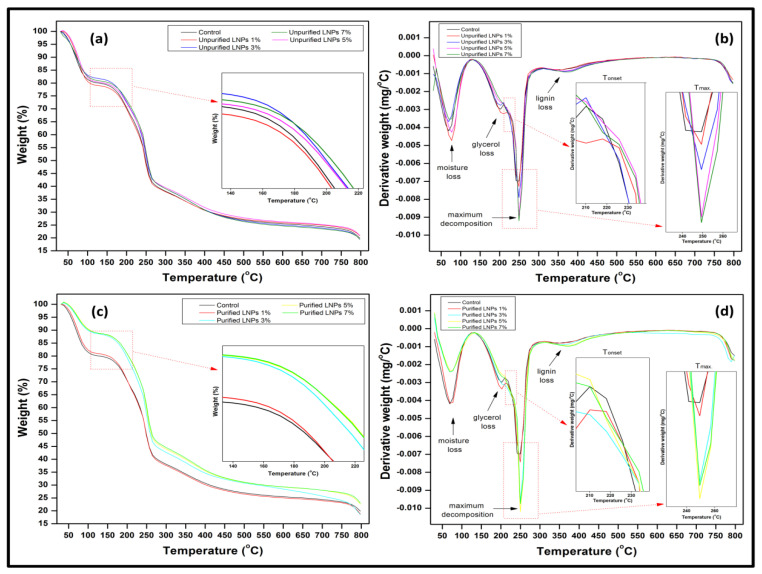
Thermogravimetric analysis of unpurified LNPs based films (**a**) TGA and (**b**) DTG, and purified LNPs based films (**c**) TGA and (**d**) DTG as a function of temperature for the LNPs/macroalgae biopolymer films.

The thermal degradation properties of bioplastic films were analyzed using thermogravimetric (TG) analysis. The results in Figure 9 present the weight loss and derivative weight loss (DTG) as a function of the pure macroalgae film’s temperature and those filled with LNPs. The TG profile of the samples containing purified LNPs except for 1% was markedly different to those of the unpurified samples and control sample, which, in turn, were practically superimposable on one another with solely negligible differences able to be observed in the overlaid TG curves. The initial stage of degradation that occurred about 40 °C to 100 °C in the films was assigned to the loss of moisture trapped within the films [43,44]. The corresponding weight losses in the purified LNPs films were considered lost, suggesting that these films had an inherently lower moisture content than unpurified LNPs films and control film. This finding was also consistent with the previous moisture content (Figure 3a), so far as purified LNPs significantly decreased the inherent moisture content of the macroalgae with an apparent downward trend in the moisture content function of purified LNPs concentration. Furthermore, the second stage in the thermal decomposition of the films observed within the temperature range of around 160 °C to 200 °C was ascribed to the maximum volatilization of glycerol (plasticizer) from the biopolymer matrix [44]. The third stage shown in the TG feature was also the main degradation stage of films that occurred at approximately 220 °C to 260 °C corresponded to the macroalgae polymer’s thermal decomposition. Finally, the fourth feature in thermal degradation at approximately 320 °C to 390 °C could be attributed to the maximum degradation of LNPs [45]. However, the film’s glycerol removal does not represent the film’s thermal stability as it was used as a plasticizer. The film is stable until the onset temperature (above 220 °C), which is that of the matrix (macroalgae).

Table 1 summarizes the numerical data derived from the TG thermograms displayed in Figure 9. The initial temperature of decomposition (T_on_) and the maximum temperature of decomposition (T_max_) were shifted to a higher temperature as the concentration of unpurified and purified LNPs increased in macroalgae films up to 5% of both LNPs and decreased after the concentrations. The onset temperature shifted from 221.52 °C to an average 229.80 °C and shifted the temperature at which the maximum rate of degradation occurred from 244.96 °C to an average of 247.55 °C. This indicated that the addition of LNPs enhanced the macroalgae films’ thermal stability up to the optimum concentration of the LNPs. A higher temperature of thermal decomposition represented better thermal stability of the material [28]. Films containing purified LNPs had better thermal stability since the T_on_ and T_max_ were higher than those with unpurified LNPs. The Ton and Tmax optimum were observed for films with 5% purified LNPs of 242.63 °C and 249.73 °C, respectively. Such improvement could be attributed to the stronger intermolecular interactions formed by hydrogen bonding between the macroalgae matrix and the nanofiller. Consequently, this required more thermal energy to break the intermolecular bonding, rendered to higher T_on_ and T_max_. Besides, dispersing agent (glycerol) could support better lignin–matrix interaction that led to an increase in thermal degradation [40]. In the context of mass loss, they decreased as the nanofiller loading increased from 1% to 7% regardless of the good adhesion and intermolecular bonding between the LNPs and the macroalgae matrix. The mass loss for films containing purified LNPs was comparatively lower than films containing unpurified LNPs and neat film. The mass loss data at 100 °C indicated that the inherent water content in the LNPs-based films was about 21% less than the control. The average mass loss of the films filled with LNPs at 250 °C was around 10% less than the control, further confirming that the LNPs-based films, especially purified LNPs, were thermally stabilized to some extent. At the extreme temperatures of 400 °C and 800 °C, the films’ average mass losses were about 3% and 2%, respectively, less than the control, thereby leading to the same conclusion.

#### 3.2.6. Color and Opacity Properties

The color and opacity of packaging film are essential properties, where consumer acceptance could be gained by a key relevance with the visual presentation of the contents. The parameters are usually performed to control the influence of light rays translucent across the film and hinder discoloration [11]. The macroalgae composite films prepared via solution casting technique were homogeneous and yellowish. As referred to in Table 2, significant changes were investigated overall as LNPs fillers loading increased. In visual observation (Table 2, right column), all-composite films were still transparent after introducing the nanofillers, and the appearance was visually well-matched to the lightness (L*). As a function of both LNPs concentration, the redness/greenness (a*) and the yellowness/blueness (b*) of these composite films were increased. The total color difference (Δ*E*) tended to decrease by an increasing proportion of both LNPs. The changing color in the macroalgae composite film was possibly due to the change of the molecule’s biological and chemical formation [46]. 

Opacity is a measurement to determine the relative transparency of a film. A low opacity value indicates the specimens that have high transparency (less opaque nature). This analysis has been described as an important parameter of film applications, especially as a packaging material. It controls the penetration of sunlight, fluorescent light, or incandescent light across the films. Such penetration can lead to discoloration, deterioration, and loss of nutrients due to photodegradation. Therefore, protection against incident light is required, particularly for products with sensitive light-catalyzed degradation reactions [22]. As shown in Table 2, composite films filled with LNPs presented higher opacity than pure macroalgae films. The opacity value increased as the loading of unpurified and purified LNPs increased, indicating a reduction of film transparency. This finding might be due to the filling up of gaps by both nanofillers that could absorb light of the wavelengths and hinder the light rays from permeating the film [47]. This finding is in great agreement with the FESEM micrographs (Figure 7). 

Overall, the addition of unpurified and purified LNPs reduced the brightness and transparency of macroalgae films. The more internal layers formed in the composite films filled with LNPs than control film may correspond to this substance. The observation in the right column of Table 2 agreed well with color and opacity analysis. A similar finding was reported by Zadeh et al. [44] with alkali lignin and lignosulfonate in enzymatically modified soy protein. Based on their color and opacity, the authors revealed that lignin was homogeneously dispersed in the matrix.

#### 3.2.7. Surface Morphology and Hydrophobicity Properties

The results of surface morphology and contact angle of macroalgae reinforced with unpurified and purified LNPs biopolymer films are presented in Table 3. The purified LNPs film surfaces’ micrographs showed a smoother and more uniform surface than unpurified LNPs and control films’ surfaces. This is more significant with the composite film added with 5% of the purified nanofiller. This indication revealed that the fillers were well-dispersed in the macroalgae matrix. As shown in the table’s surface morphology column, the incorporation of LNPs in a higher concentration (7%) led to the formation of rougher surfaces and voids of bioplastic films. This phenomenon can reduce the hydrophobicity and water barrier properties of the films. One advanced indicator of the structural integrity between matrix and filler is homogeneity, enhancing the mechanical properties and thermal stability. The absence of impurities of the purified LNPs films corresponds to more organized layers than the films containing LNPs without purification process, resulting in improved water barrier properties. Otherwise, the surface of unpurified LNPs-based films was rougher and coarser. Lower interfacial interaction between macroalgae and unpurified LNPs was responsible for their weaker bonding that was indicated with the holes. It was also observed the gaps in the control film surface that led to the brittleness. This indication was similar to its micrograph of fracture surface (Figure 7).

The contact angle is the converse measure of the wettability of polymers. The macroalgae biopolymer films have been reported with hydrophilic properties. The incorporation of both LNPs significantly increased (*p* < 0.05) the prepared films’ contact angle value compared to their control film. The enhancement of contact angle followed by the addition of LNPs might occur due to the strong intermolecular hydrogen bonding between LNPs and hydroxyl groups in macroalgae. Consequently, this type of formation generated a reduction of free available hydroxyl groups in the matrix to link to the surrounding water molecules, thereby reducing the hydrophilicity of the composite films [48]. Among all the films, the composite films with 5% of purified LNPs had the optimum contact angle value, which reduced the water contact with the film surface and established a solid water droplet. This result showed that the nanofillers’ interfacial adhesion and the macroalgae could probably be enhanced as the LNPs loading increased up to the threshold.

Interestingly, the films’ contact angle filled with purified LNPs was higher than films incorporated with unpurified LNPs. This finding stated that the purification of LNPs increased its miscibility and compatibility within the macroalgae. As a result, the purified nanofillers’ introduction reduced the film’s porosity and provided more organized-surfaces with lesser clefts.

## 4. Conclusions

Macroalgae-based bioplastic films incorporated with unpurified LNPs and purified LNPs were successfully developed and characterized. A noticeable enhancement in the physical, mechanical, morphological, structural, thermal, optical, and water barrier properties of the macroalgae-based films was presented by incorporating both nanofillers. As a function of both LNPs concentration, the MC, MAC, TS, YM, EAB, surface morphology, and contact angle properties were significantly enhanced. However, bioplastic films with purified LNPs exhibited superior properties compared to unpurified LNPs-based bioplastic films. The properties of unpurified LNPs and purified LNPs in macroalgae are different due to miscibility and compatibility. This was influenced by the particle size, surface charge of particles, and reduction of free available hydroxyl groups. Herein, the optimum performance was provided by a composite film containing 5% purified LNPs, which resulted in 36.70 MPa, 343.59 MPa, 44.76%, and 96.83° of TS, YM, EAB, and contact angle, respectively. Since this study’s bioplastic films showed considerable functional properties such as mechanical, thermal, and water barriers, they could be a good candidate to replace conventional petroleum-derived plastics in packaging material for a wide range of applications. A more detailed investigation of the functioned LNPs into the macroalgae matrix is essential in future work to achieve more enhancements.

## Figures and Tables

**Table 1:** Decomposition temperatures and mass loss data of macroalgae film with different loading of unpurified LNPs and purified LNPs.

Filler Loading (%)	Decomposition Temperature (°C)		Mass Loss (%)			
	T_onset_	T_max._	100 °C	250 °C	400 °C	800 °C
Control	221.52	244.96	19.43	51.35	69.73	81.42
Unpurified lignin						
1	222.39	246.14	18.64	50.78	69.19	81.23
3	223.71	246.37	17.82	47.86	69.01	80.61
5	235.13	248.33	17.43	46.72	68.94	80.12
7	224.12	247.85	16.94	46.14	68.04	79.13
Purified lignin						
1	224.03	247.15	17.77	50.31	69.14	80.57
3	235.29	248.48	10.17	42.16	65.25	79.08
5	242.63	249.73	9.79	40.01	64.39	77.40
7	239.37	248.95	9.58	38.83	64.07	76.99
Average	229.80	247.55	15.29	46.02	67.53	79.62
Difference			21%	10%	3%	2%

**Table 2:** Colour and opacity of macroalgae-based biopolymer film reinforced with unpurified and purified LNPs.

Filler Loading (%)	L*	a*	b*	Δ*E*	Opacity	Photograph
Control	85.15 ± 0.12 ^f^	0.29 ± 0.01 ^a^	5.56 ± 0.01 ^a^	85.33 ± 0.12 ^g^	0.91 ± 0.05 ^a^	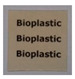
Unpurified lignin						
1	74.99 ± 0.55 ^e^	3.42 ± 0.12 ^b^	16.33 ± 0.19 ^c^	76.82 ± 0.50 ^f^	3.35 ± 0.09 ^b^	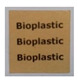
3	59.06 ± 1.39 ^c^	11.24 ± 0.66 ^d^	24.51 ± 0.04 ^g^	64.92 ± 1.14 ^d^	6.42 ± 0.06 ^d^	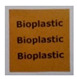
5	51.01 ± 0.90 ^b^	15.01 ± 0.38 ^e^	22.21 ± 0.50 ^f^	57.62 ± 0.88 ^b^	7.33 ± 0.10 ^e^	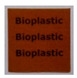
7	41.66 ± 0.45 ^a^	17.26 ± 0.04 ^f^	13.64 ± 0.50 ^b^	47.12 ± 0.53 ^a^	8.82 ± 0.16 ^f^	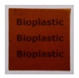
Purified lignin						
1	69.65 ± 1.09 ^d^	5.64 ± 0.52 ^c^	19.82 ± 0.81 ^d^	72.64 ± 0.78 ^e^	4.76 ± 0.16 ^c^	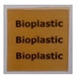
3	56.57 ± 0.46 ^c^	11.40 ± 0.18 ^d^	21.87 ± 0.17 ^ef^	61.71 ± 0.39 ^c^	6.61 ± 0.06 ^d^	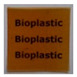
5	49.74 ± 1.45 ^b^	14.65 ± 0.57 ^e^	20.34 ± 0.66 ^de^	55.71 ± 1.39 ^b^	7.62 ± 0.15 ^e^	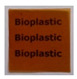
7	42.41 ± 0.93 ^a^	16.73 ±0.11 ^f^	14.35 ± 1.25 ^b^	47.81 ± 1.15 ^a^	8.68 ± 0.18 ^f^	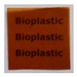

Values are presented as the mean with one standard deviation error bar. The letters (a, ab, abc, b, bc, bcd, cd, cde, d, de, e) above data bars indicate no significant difference in the values (*p* < 0.05) analysed by ANOVA test.

**Table 3:** The FESEM images of surface morphology and contact angle properties of macroalgae-based biopolymer film reinforced unpurified and purified LNPs.

Filler Loading (%)	Unpurified Lignin			Purified Lignin		
	Surface Morphology	Droplet Image	Contact Angle of Films (θ)	Surface Morphology	Droplet Image	Contact Angle of Films (θ)
Control	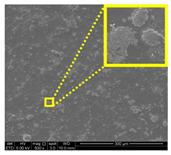	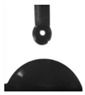	67.46^o^ ± 0.19 ^a^ 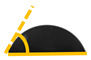	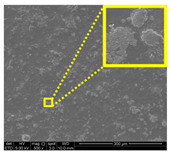	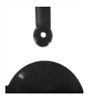	67.46^o^ ± 0.19 ^a^ 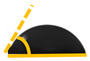
1	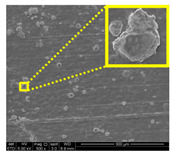	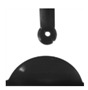	73.07^o^ ± 0.42 ^b^ 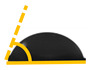	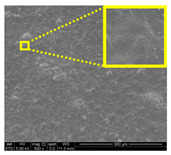	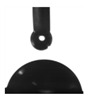	78.27^o^ ± 0.34 ^d^ 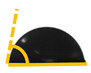
3	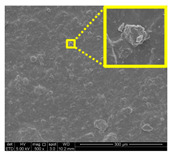	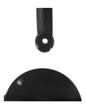	75.59^o^ ± 0.70 ^c^ 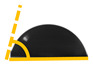	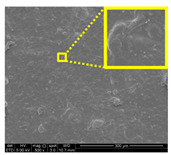	^ 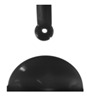 ^	90.80^o^ ± 0.12 ^g^ 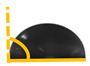
5	^ 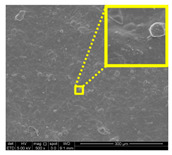 ^	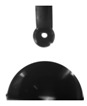	87.45^o^ ± 0.31 ^f^ 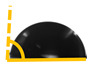	^ 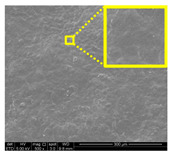 ^	^ 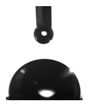 ^	96.83^o^ ± 0.40 ^h^ 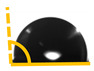
7	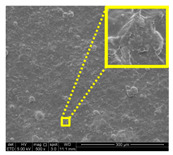	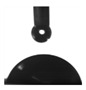	74.89^o^ ± 0.48 ^c^ 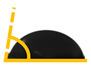	^ 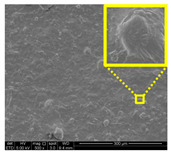 ^	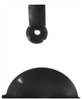	84.51^o^ ± 0.26 ^e^ 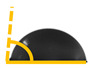

Values are plotted as the mean with one standard deviation error bar. Means in the same columns of contact angle followed by different superscript letters indicate significant differences (*p* < 0.05).

## Data Availability

Not applicable.
